# Post-traumatic stress disorder (PTSD), anger and mental health of school students in Syria after nine years of conflict: a large-scale school-based study

**DOI:** 10.1192/bjo.2021.695

**Published:** 2021-06-18

**Authors:** Sami Jomaa, Ameer Kakaje, Ragheed Al Zohbi, Ayham Alyousbashi, Rawan N K Abdelwahed, Osama Hosam Aldeen, Mohammad Marwan Alhalabi, Ayham Ghareeb, Youssef Latifeh

**Affiliations:** 1 Faculty of Medicine, Damascus University; 2 Faculty of Medicine, Aleppo University; 3 Psychiatry Department, Al-Mowasat University Hospital, Department of Internal Medicine, Syrian Private University

## Abstract

**Aims:**

The Syrian crisis has entered its ninth year with many being affected by the war. This is the largest-scale study that aims to evaluate the psychological profile of secondary school students in Syria.

**Method:**

This is a cross-sectional study in schools in Damascus, Syria. The surveys assessed working habits, smoking, war exposure, grades, socioeconomic status (SES), social support, health-related quality of life (HRQL), post-traumatic stress disorder (PTSD), problematic anger, and other parameters.

**Result:**

This study included 1369 students of which 53% suffered from PTSD and 62% from problematic anger. Around 46% declared a fair or worse general health and 61% had moderate or severe mental health. Only 9.3% did not report exposure to any war-related variable. War exposure had an impact on PTSD, anger, and HRQL, but not on students' grades. Smoking, having consanguineous parents, and working did not have a clear association with grades or anger. Social support weakly reduced PTSD and anger scores. Interestingly, working was associatedwith lower PTSD scores but was associated with a worse physical component of HRQL.

**Conclusion:**

This is the largest study on school students in Syria that reports the psychological ramifications of war. Although the direct effects of war could not be precisely described, the high burden of PTSD and anger distress was a strong reflection of the chronic mental distress.

